# rRGD3^mu^, a Triple-RGD Recombinant Peptide, Suppresses Malignant Phenotypes in Nasopharyngeal Carcinoma-Associated Models Through the Modulation of ITGB1-Associated FAK/AKT Signaling

**DOI:** 10.3390/ijms27115045

**Published:** 2026-06-03

**Authors:** Qianhui Yuan, Fuxin Zhou, Xiaotong Li, Jingyu Zhang, Yuebin Zhang, Jihong Yao, Mei Lv, Jihong Wang, Li Lv

**Affiliations:** 1 Department of Pharmacology, Dalian Medical University, Dalian 116044, China; yuanqianhui1202@outlook.com (Q.Y.); z18053477872@outlook.com (F.Z.); lch15042431080@live.com (X.L.); yaojihong65@hotmail.com (J.Y.); 2 School of Life Sciences, Liaoning Normal University, Dalian 116081, China; jingyuzhang2000@hotmail.com (J.Z.); yuebinz@hotmail.com (Y.Z.); 3 Department of Otorhinolaryngology and Head and Neck Surgery, Dalian Medical University, Dalian 116100, China; lv_mei@163.com

**Keywords:** nasopharyngeal carcinoma, rRGD3^mu^, recombinant peptide, integrin β1, FAK/AKT axis, apoptosis, EMT

## Abstract

Nasopharyngeal carcinoma-associated malignant epithelial models remain useful for exploring integrin-related therapeutic strategies. In this study, we evaluated the antitumor activity and potential mechanisms of rRGD3^mu^, a recombinant peptide with a triple-RGD architecture. Using CNE2 cells as the primary experimental model, we evaluated cell viability, colony formation, migration, invasion, adhesion, apoptosis-related marker expression, and EMT-associated molecular changes. In vivo efficacy was assessed using a CNE2 cell-derived BALB/c nude mouse xenograft model. rRGD3^mu^ inhibited CNE2 cell viability, clonogenic growth, migration, invasion, and adhesion in a dose-dependent manner and suppressed xenograft tumor growth under the tested dosing schedule. Mechanistically, rRGD3^mu^ promoted mitochondria-associated apoptosis, as indicated by an increased Bax/Bcl-2 ratio and caspase-9/3 activation, and modulated the expression of EMT-associated markers, including E-cadherin, N-cadherin, vimentin, and MMP2. Bioinformatic analysis and experimental validation suggested that ITGB1-containing integrin complexes might serve as important mediators and putative cellular engagement sites of rRGD3^mu^. rRGD3^mu^ treatment reduced ITGB1 protein abundance and attenuated FAK/AKT signaling. ITGB1 knockdown partially mimicked the effects of rRGD3^mu^ and reduced the additional cellular response to rRGD3^mu^ treatment, supporting the substantial contribution of ITGB1-associated signaling. These findings provide preliminary mechanistic evidence that rRGD3^mu^ suppresses malignant phenotypes in CNE2-based models, at least in part through modulation of ITGB1-associated FAK/AKT signaling.

## 1. Introduction

Nasopharyngeal carcinoma (NPC), a highly aggressive squamous cell malignancy [1], has a distinct geographical distribution, with a notably high prevalence in East and Southeast Asia [2,3]. While NPC is traditionally sensitive to radiotherapy and chemotherapy, long-term outcomes remain constrained by late-stage diagnosis and a formidable propensity for local recurrence and distant metastasis [4,5]. Despite advancements in multimodal therapeutic strategies, a significant subset of patients inevitably develops treatment resistance. The insidious progression of NPC, characterized by extensive systemic invasion, underscores an urgent clinical imperative to delineate the molecular mechanisms driving malignancy and to develop innovative, highly efficacious therapeutic agents capable of simultaneously arresting tumor proliferation and metastatic dissemination [6].

Peptides and proteins harboring the Arg-Gly-Asp (RGD) motif are promising therapeutic candidates because of their high affinity for integrins [7], which critically regulate tumor angiogenesis and metastasis [8]. Drawing inspiration from the structural diversity of natural bioactive proteins, we previously developed and validated a triple-RGD architecture as an effective scaffold for tumor inhibition [9]. To optimize its translational potential, we bioengineered rRGD3^mu^, a novel trivalent recombinant peptide (63 amino acids, 7.83 kDa) derived from a structurally refined protein template. Unlike traditional monovalent cyclic RGD peptides (e.g., cilengitide), which often suffer from suboptimal binding avidity, rRGD3^mu^ incorporates a triple-RGD architecture engineered to leverage receptor clustering, thus significantly enhancing its multivalent avidity for integrin receptors. While this truncated design strategically bridges the gap between small-molecule RGD mimetics and full-length proteins by potentially improving tissue penetration while possibly reducing immunogenic risk compared with larger protein scaffolds [10,11], the precise molecular mechanisms and the receptor subtypes and signaling pathways contributing to its biological activity in NPC-related models remain to be fully elucidated.

Integrins constitute a dominant family of transmembrane adhesion receptors that physically and functionally tether the actin cytoskeleton to the extracellular matrix (ECM) [12,13,14]. Integrin β1 (ITGB1) has emerged as a critical oncogenic driver that coordinates tumor cell survival and aggressive progression [15,16,17]. Upon interaction with ECM components, ITGB1 facilitates the recruitment and activation of focal adhesion kinase (FAK), subsequently driving the downstream AKT signaling cascade [18,19,20]. Although the ITGB1/FAK/AKT axis is a fundamental driver of apoptotic evasion and epithelial−mesenchymal transition (EMT) in NPC, achieving functional selectivity for specific integrin subunits remains a major pharmacological challenge. Consequently, identifying novel antagonists that can effectively perturb this pathway offers a promising strategy to counteract NPC malignancy.

Building upon these considerations, we hypothesized that rRGD3^mu^ may exert its antitumor activity, at least in part, by modulating ITGB1-associated signaling. In the present study, we systematically evaluated the pharmacological efficacy of rRGD3^mu^ in both in vitro and in vivo NPC models. Our findings demonstrate that rRGD3^mu^ has potent antiproliferative and antimetastatic effects. By integrating bioinformatics screening with functional assays, we identified ITGB1 as a pivotal mediator of NPC malignancy and further substantiated that rRGD3^mu^ effectively attenuates the FAK/AKT axis in an ITGB1-dependent manner. By characterizing the attenuation of malignant phenotypes through the modulation of ITGB1/FAK/AKT signaling axis, this study highlights the potential of rRGD3^mu^ as a targeted therapeutic candidate for NPC intervention.

## 2. Results

### 2.1. Characterization and Purification of the Recombinant Peptide rRGD3^mu^

**Figure 1:**
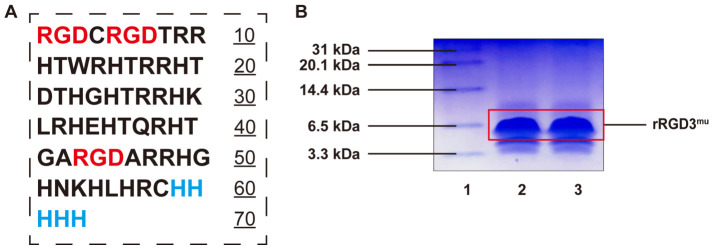
Characterization and purification of the recombinant peptide rRGD3^mu^. (**A**) Amino acid sequence of the rRGD3^mu^ peptide; the RGD motifs are highlighted in red, the His-tag is highlighted in blue. (**B**) Preliminary electrophoretic profiling of the purified rRGD3^mu^ preparation by Tricine-SDS−PAGE. Lane 1: markers; Lanes 2 and 3: purified rRGD3^mu^ protein.

rRGD3^mu^ is a bioengineered recombinant peptide designed on the basis of a structural template derived from *Lampetra japonica* oral gland secretions and incorporates a trivalent RGD motif (Figure 1A). The purified recombinant peptide preparation was analyzed by tricine-SDS−PAGE to obtain a preliminary electrophoretic profile. A predominant band migrating at approximately 7.8 kDa was observed, which is consistent with the calculated molecular mass of rRGD3^mu^ (Figure 1B). A faint secondary band was also detected slightly below the major band. This pattern may be related to anomalous electrophoretic mobility commonly observed for small, truncated, or highly charged peptides under denaturing conditions, although minor peptide-related species, degradation products, or residual impurities cannot be excluded by SDS−PAGE alone. These results suggest that rRGD3^mu^ was the predominant component detected under the electrophoretic conditions used.

### 2.2. rRGD3^mu^ Suppresses the Malignant Phenotypes of CNE2 Cells In Vitro

**Figure 2:**
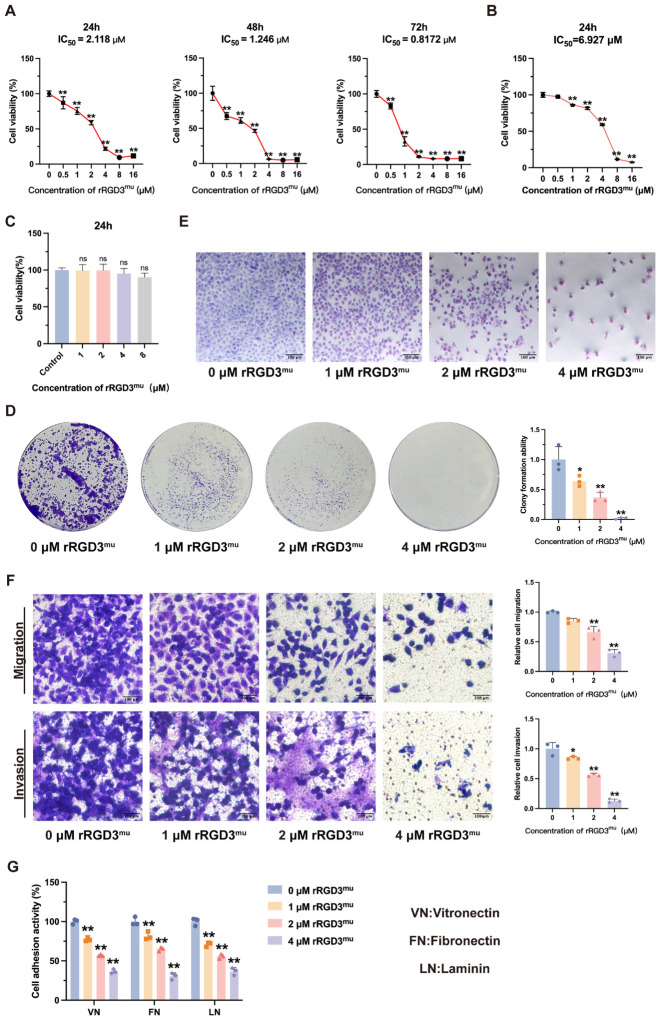
rRGD3^mu^ suppresses the malignant phenotypes of CNE2 cells in vitro. (**A**) Dose- and time-dependent inhibitory effects of rRGD3^mu^ on CNE2 cells, as measured by a CCK-8 assay (*n* = 6). (**B**) Viability of HNE1 cells, as measured by a CCK-8 assay (*n* = 6). (**C**) Cytotoxicity of rRGD3^mu^ in normal NP-69 cells (*n* = 6). (**D**) Clonogenic potential assessed via a colony formation assay (*n* = 3). (**E**) Morphological alterations visualized by Giemsa staining (*n* = 3). (**F**) Cell migratory and invasive capabilities evaluated via Transwell assays (*n* = 3). (**G**) Adhesion to ECM components (*n* = 3). Scale bars: 100 μm. Significance is indicated as * *p* < 0.05 and ** *p* < 0.01 compared with the control group.

The anti-proliferative efficacy of rRGD3^mu^ was evaluated in the NPC cell lines CNE2 and HNE1. rRGD3^mu^ exhibited robust, dose- and time-dependent inhibitory effects on CNE2 cells (Figure 2A), with IC_50_ values of 2.118, 1.246, and 0.8172 µM at 24, 48, and 72 h, respectively. CCK-8 assays revealed that rRGD3^mu^ dose-dependently inhibited the viability of HNE1 cells, with an IC_50_ value of approximately 6.927 µM at 24 h (Figure 2B). This responsiveness gradient reflects the molecular heterogeneity of NPC, while both lines remain within a potent low-micromolar window. Owing to its increased sensitivity, CNE2 cells were selected as the primary model for subsequent mechanistic and in vivo investigations. In parallel, NP-69 cells showed lower sensitivity to rRGD3^mu^ within the tested concentration range (Figure 2C), suggesting a preliminary differential response between malignant epithelial cells and immortalized nasopharyngeal epithelial cells. The clonogenic potential of CNE2 cells was markedly compromised upon rRGD3^mu^ exposure (Figure 2D), with nearly total suppression at a concentration of 4 µM.

Morphological assessment via Giemsa staining (Figure 2E) further corroborated these cytotoxic effects, revealing a progressive reduction in cell density and characteristic morphological distortions in rRGD3^mu^-treated cells. To evaluate the effect of rRGD3^mu^ on metastatic potential, Transwell assays (Figure 2F) were performed. The results indicated that rRGD3^mu^ dramatically attenuated the migratory and invasive capacities of CNE2 cells in a concentration-dependent manner. Four-hour cell adhesion assays revealed that rRGD3^mu^ reduced the attachment of CNE2 cells to ECM components, including VN, FN, and LN, suggesting that rRGD3^mu^ can interfere with early integrin-associated cell–ECM attachment (Figure 2G). However, because the migration and invasion assays (Figure 2F) were performed over 24 h at concentrations that also reduced cell viability, the observed decreases in migrated and invaded cells should be interpreted as cumulative effects of impaired cell–ECM interaction, reduced motile capacity of the remaining viable cells, and rRGD3^mu^-induced cytotoxicity. Collectively, these data indicate that rRGD3^mu^ suppresses multiple malignant phenotypes of CNE2 cells in vitro, but the migration and invasion results should be interpreted considering its concurrent cytotoxic effects.

### 2.3. rRGD3^mu^ Suppresses the Growth of CNE2 Cell-Derived Xenografts In Vivo

**Figure 3:**
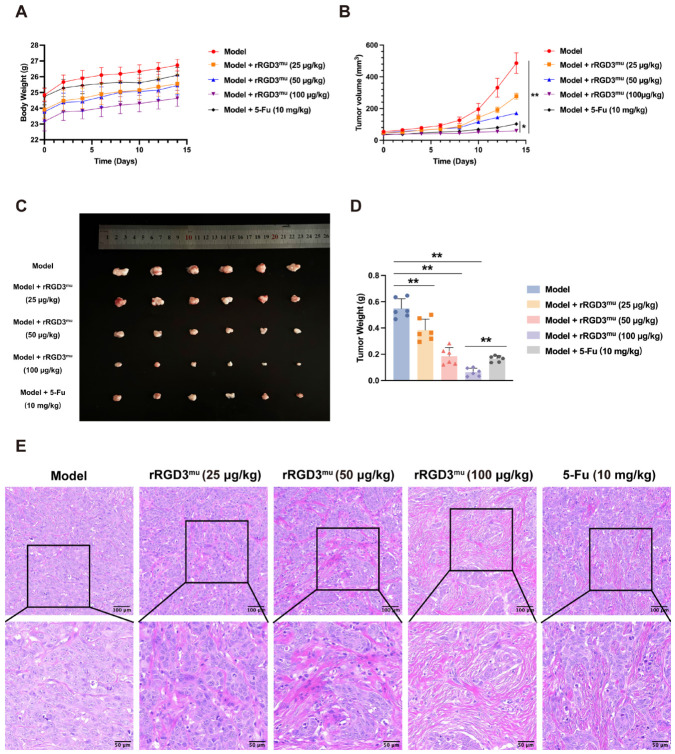
rRGD3^mu^ suppresses the growth of CNE2 cell-derived xenografts in vivo. (**A**) Monitoring of body weight throughout the treatment course. (**B**) Tumor growth curves based on subcutaneous tumor volume measurements. (**C**) Macroscopic appearance of tumors harvested from different cohorts upon study termination. (**D**) Comparative analysis of final subcutaneous tumor weights. (**E**) Histopathological evaluation of tumor sections via H&E staining. Scale bars: 100 μm and 50 μm. Significance is indicated as * *p* < 0.05 and ** *p* < 0.01 compared with the model group.

To systematically evaluate the in vivo antitumor potential of rRGD3^mu^, we generated a subcutaneous xenograft model in BALB/c nude mice. No significant fluctuations in body weight were observed among the groups (Figure 3A); the absence of significant body weight loss suggested that no overt systemic toxicity occurred under the tested dosing schedule. By day 14, the mean tumor diameter in the model group reached 11.93 ± 1.56 mm, whereas it was dose-dependently reduced to 10.57 ± 2.23 mm, 8.47 ± 0.63 mm, and 5.90 ± 0.16 mm in the 25, 50, and 100 µg/kg rRGD3^mu^-treated groups, respectively (*p* < 0.01 for the high-dose group). Under the tested dosing schedule, compared with the 5-Fu group, the 100 µg/kg rRGD3^mu^ group had a smaller mean tumor diameter (6.63 ± 0.53 mm). Consistent with the diameter measurements, both tumor volume and tumor weight progressively decreased with increasing rRGD3^mu^ dosage (Figure 3B–D).

Histopathological evaluation via H&E staining (Figure 3E) revealed extensive necrotic regions and acellular areas in rRGD3^mu^-treated tumors, in contrast to the densely packed neoplastic cells and intact stromal architecture in the model group. These histological changes are consistent with reduced tumor viability and increased necrotic changes in rRGD3^mu^-treated xenografts.

### 2.4. rRGD3^mu^ Promotes Apoptosis and Modulates the Expression of EMT-Associated Markers in CNE2 Cells by Attenuating FAK/AKT Signaling

**Figure 4:**
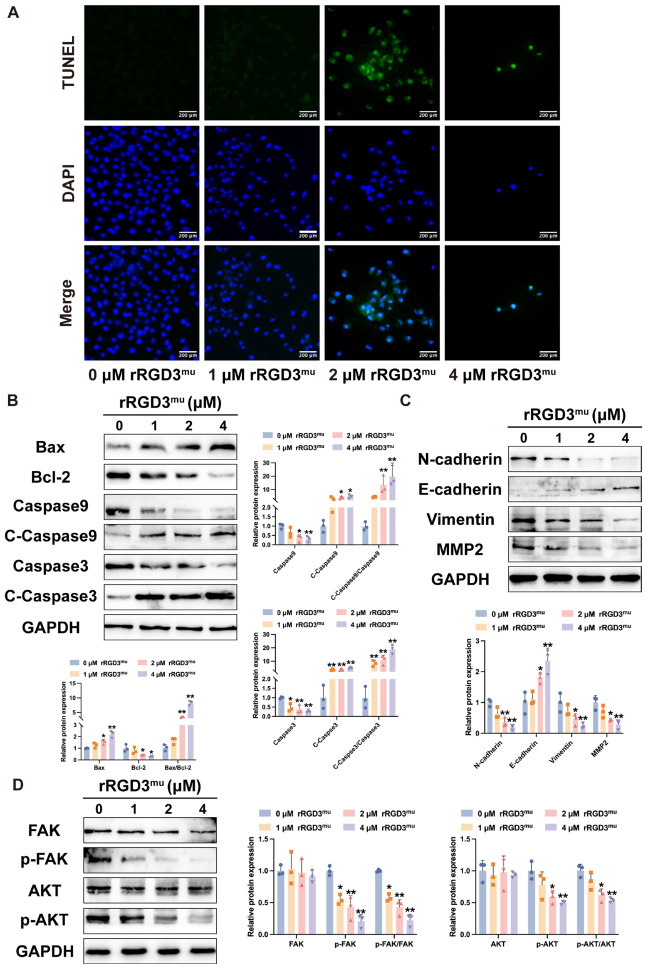
rRGD3^mu^ promotes apoptosis and modulates the expression of EMT-associated markers in CNE2 cells, with attenuation of FAK/AKT signaling. (**A**) TUNEL assay showing the induction of apoptosis in CNE2 cells (n = 3). Scale bar = 200 μm. (**B**–**D**) Western blot analysis of apoptosis-related proteins (**B**), EMT markers (**C**), and FAK/AKT signaling components (**D**) in CNE2 cells treated with rRGD3^mu^. For all Western blot panels, Lanes 1–4 correspond to rRGD3^mu^ concentrations of 0, 1, 2, and 4 μM (n = 3). Significance is indicated as * *p* < 0.05 and ** *p* < 0.01 compared with the control group.

TUNEL staining revealed that rRGD3^mu^ administration markedly increased the frequency of apoptotic cells (Figure 4A). Western blot analysis demonstrated that rRGD3^mu^ treatment attenuated Bcl-2 protein expression but increased Bax levels (Figure 4B), thus resulting in a marked increase in the Bax-to-Bcl-2 ratio. This imbalance subsequently triggered proteolytic activation of the caspase cascade, as evidenced by the markedly elevated expression levels of cleaved caspase-9 and cleaved caspase-3. These findings substantiate that rRGD3^mu^ initiates a mitochondria-dependent apoptotic pathway characterized by caspase activation.

Western blot analysis revealed that rRGD3^mu^ modulated the expression of EMT-associated markers, as indicated by increased E-cadherin expression and decreased N-cadherin, vimentin, and MMP2 levels (Figure 4C). These molecular changes are consistent with the attenuation of EMT-associated features. Together with the reduced migration and invasion observed in the Transwell assays, these findings suggest that rRGD3^mu^ attenuates EMT-associated molecular and functional features. However, because migration and invasion assays were performed over 24 h at concentrations that also affected viability, these phenotypic effects should be interpreted as combined consequences of impaired adhesion, reduced motility, and cytotoxicity. Furthermore, rRGD3^mu^ administration induced a substantial decrease in the phosphorylation levels of FAK and AKT (Figure 4D), whereas the total protein abundance of these kinases remained unaffected. These data suggest that rRGD3^mu^ exerts dual regulatory effects on apoptosis and EMT by effectively attenuating the activation of the FAK/AKT signaling axis.

### 2.5. ITGB1-Containing Integrin Complexes Function as Important Mediators and Putative Cellular Engagement Sites of rRGD3^mu^ in CNE2 Cells

**Figure 5:**
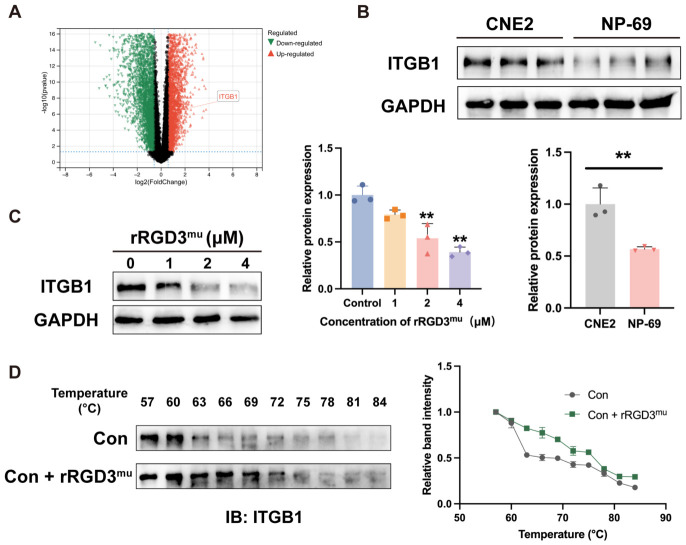
ITGB1-containing integrin complexes function as important mediators and putative cellular engagement sites of rRGD3^mu^ in CNE2 cells. (**A**) Bioinformatics analysis of ITGB1 expression in the TCGA-HNSC cohort (n = 566) using the Limma algorithm. (**B**) Western blot analysis comparing ITGB1 expression levels between CNE2-derived malignant epithelial cells and NP-69 cells. (**C**) Western blot analysis of ITGB1 expression in CNE2 cells following rRGD3^mu^ treatment at concentrations of 0, 1, 2, and 4 μM (n = 3). (**D**) CETSA analysis of endogenous ITGB1 thermal stability after rRGD3^mu^ treatment. CNE2 cells were treated with rRGD3^mu^ (2 µM) for 2 h before heating. Representative Western blot bands (**left**) and the corresponding thermal melting curves (**right**) demonstrate that rRGD3^mu^ significantly increased the thermal stability of ITGB1. Significance is indicated as ** *p* < 0.01 compared with the control group.

To evaluate the clinical relevance of ITGB1 and identify the potential molecular targets of rRGD3^mu^ in NPC, we leveraged the TCGA-HNSC cohort using the R-based Limma algorithm. The results revealed that ITGB1 is significantly overexpressed in malignant tumor tissues compared with adjacent non-neoplastic tissues (Figure 5A), suggesting its involvement in the malignant progression of head and neck squamous cell carcinomas. In parallel, Western blot analysis confirmed that, compared with that in immortalized human NP-69 cells, ITGB1 expression was markedly elevated in CNE2 NPC cells (Figure 5B). Crucially, rRGD3^mu^ treatment induced a dose-dependent decrease in the level of the ITGB1 protein in CNE2 cells (Figure 5C). To evaluate whether rRGD3^mu^ treatment affects the thermal stability of endogenous ITGB1-containing complexes in cells, CETSA was performed. ITGB1 in the control group began to denature at 63 °C, whereas rRGD3^mu^ treatment significantly increased its thermal stability, rendering the protein resistant to thermal degradation up to 69 °C (Figure 5D). These findings support cellular target engagement involving endogenous ITGB1-containing integrin complexes and suggest that ITGB1-associated signaling may contribute to the biological response to rRGD3^mu^.

### 2.6. ITGB1 Contributes to rRGD3^mu^-Induced Antitumor Responses in CNE2 Cells

**Figure 6:**
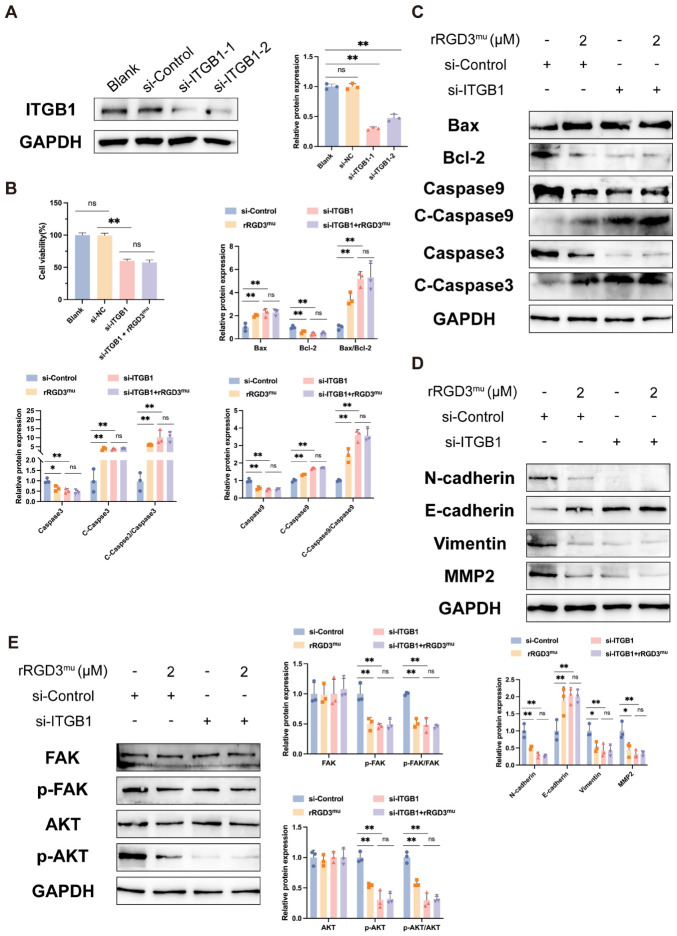
ITGB1 contributes to rRGD3^mu^-induced antitumor responses in CNE2 cells. (**A**) Western blot analysis of ITGB1 protein levels in cells transfected with si-Control or ITGB1-specific siRNAs (si-ITGB1-1/2). (**B**) Cell viability was measured by a CCK-8 assay. CNE2 cells were transfected with si-ITGB1 and subsequently treated with rRGD3^mu^ at 2 μM. Blank: nontransfected cells. (**C**–**E**) In all panels, Lanes 1–4 correspond to the following groups: si-Control, si-Control + rRGD3^mu^, si-ITGB1, and si-ITGB1 + rRGD3^mu^, respectively (n = 3). Western blot analysis of apoptosis-related proteins (**C**), EMT markers (**D**), and FAK/AKT signaling components (**E**) in CNE2 cells following ITGB1 knockdown with or without rRGD3^mu^ treatment at 2 μM. Significance is indicated as * *p* < 0.05 and ** *p* < 0.01 for the indicated comparisons.

To assess whether ITGB1 contributes to rRGD3^mu^-induced antitumor responses in CNE2 cells, siRNA-mediated knockdown was performed. Two independent ITGB1-targeting siRNAs, si-ITGB1-1 and si-ITGB1-2, were used to evaluate knockdown specificity at the protein expression level. Both siRNAs reduced ITGB1 protein expression, with si-ITGB1-1 showing stronger and more consistent knockdown efficiency (Figure 6A); therefore, si-ITGB1-1 was selected for subsequent functional assays.

CCK-8 assays revealed that, compared with the blank and si-Control groups, the ITGB1-silenced group had reduced CNE2 cell viability. The viabilities of the blank and si-Control cells were comparable, indicating that the transfection procedure itself did not cause obvious cytotoxicity under these conditions. When rRGD3^mu^ was added after ITGB1 knockdown, no statistically significant further reduction in cell viability was observed compared with that in cells with ITGB1 knockdown alone (Figure 6B). These results suggest that ITGB1 depletion attenuates the additional cytotoxic response to rRGD3^mu^. However, a potential floor effect caused by reduced baseline viability after ITGB1 silencing cannot be completely excluded.

Consistent with these phenotypic changes, ITGB1 knockdown affected apoptosis-related proteins, EMT markers, and the FAK/AKT signaling axis (Figure 6C–E). The additional regulatory effects of rRGD3^mu^ on these signaling molecules were reduced after ITGB1 knockdown. Together with the CETSA-based target-engagement data, these findings support a substantial contribution of ITGB1-containing integrin complexes to rRGD3^mu^-induced antitumor responses in CNE2 cells, rather than providing an exclusive or strictly ITGB1-dependent mechanism.

## 3. Discussion

**Figure 7:**
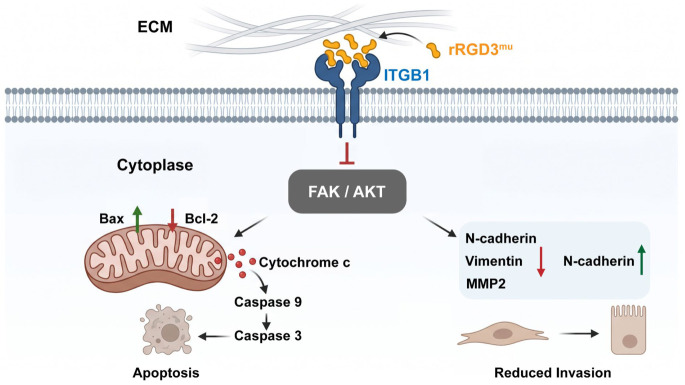
Schematic of the molecular mechanism by which rRGD3^mu^ suppresses NPC progression by attenuating the activity of the ITGB1/FAK/AKT signaling cascade.

In the present study, we successfully developed rRGD3^mu^, a novel bioengineered recombinant peptide, and evaluated its preliminary antitumor activity and effects on migration/invasion-associated phenotypes in CNE2-based models through both in vitro and in vivo experimental paradigms. Our results demonstrated that rRGD3^mu^ effectively suppressed cell viability, impaired clonogenic potential, and attenuated the invasive capacity of NPC cells. Mechanistically, this study provides evidence that the pharmacological activity of rRGD3^mu^ is closely associated with the downregulation of ITGB1 expression and the attenuation of ITGB1-related signaling. By modulating ITGB1-mediated signaling, rRGD3^mu^ attenuates the activation of the FAK/AKT signaling axis, thus contributing to mitochondria-associated apoptosis and the modulation of EMT-related molecular features (Figure 7). These findings not only characterize rRGD3^mu^ as a promising therapeutic candidate but also establish a compelling mechanistic foundation for targeting the ITGB1/FAK/AKT axis in the therapeutic intervention of NPC.

The rational design of rRGD3^mu^ may offer potential pharmacological features distinct from those of conventional monovalent RGD-based molecules. Derived from bioactive protein templates of *Lampetra japonica*, rRGD3^mu^ features a triple-RGD architecture designed to exploit the multivalent binding effect. Unlike clinical-stage monovalent RGD mimetics (e.g., cilengitide), which often suffer from suboptimal receptor avidity, this configuration enhances multivalent avidity and inhibitory potency by promoting stable receptor occupancy [21,22]. From a pharmacokinetic perspective, the refined 63-amino acid design (7.83 kDa) is strategically significant; this low molecular weight facilitates penetration through the dense stromal matrix characteristic of NPC and may facilitate tissue accessibility compared with that of larger globular proteins, although biodistribution studies are needed to verify this possibility [23]. Future investigations, including radionuclide labeling for real-time micro-SPECT/CT [24,25], are warranted to quantitatively delineate the specific in vivo biodistribution and clearance of rRGD3^mu^.

Furthermore, such a truncated framework is expected to mitigate potential immunogenicity [26], a common limitation of high-molecular-weight biologics. Crucially, the absence of significant body weight fluctuations in our in vivo model preliminarily demonstrates that rRGD3^mu^ did not induce overt systemic toxicity under the tested dosing schedule [27]. To further establish its clinical safety margin, comprehensive hematological and biochemical profiling, in addition to systemic toxicity assessments in appropriate higher-order animal models when necessary, are warranted in subsequent studies [28]. These attributes collectively bestow rRGD3^mu^ with a competitive edge in the developmental landscape of peptide-based therapeutics.

Apoptotic evasion and EMT are interconnected hallmarks that drive NPC progression [29,30,31]. Our findings reveal that rRGD3^mu^ exerts dual regulatory effects on these processes. Mechanistically, rRGD3^mu^ triggers a significant shift in the Bax/Bcl-2 ratio, initiating the intrinsic mitochondrial pathway and culminating in the Caspase-9/3 proteolytic cascade [32,33,34]. Concurrently, the modulation of EMT-associated markers—as evidenced by E-cadherin upregulation and the downregulation of N-cadherin, vimentin, and MMP2—indicates a shift toward a less mesenchymal marker profile [35,36,37]. Because the adhesion assay was performed over a shorter 4 h period than the 24 h migration and invasion assays, the adhesion results may reflect an early effect on cell–ECM interactions. This temporal pattern suggests that, early on, rRGD3^mu^ perturbs integrin–ECM engagement, which subsequently synergizes with its later-stage pro-apoptotic signaling at 24 h. This integrated cascade effectively targets both cell survival and metastatic competence, rather than the inhibition of motility being merely an artifact of cell death. The synchronization of these processes is biologically significant, as the FAK/AKT axis serves as a signaling node that integrates survival and motility cues [38]. By attenuating this axis, rRGD3^mu^ not only eliminates malignant cells but also diminishes the invasive potential of surviving cells, offering a bimodal strategy to counteract NPC growth and dissemination.

To further elucidate the molecular mechanisms underlying this therapeutic outcome, we focused on FAK, a pivotal nonreceptor tyrosine kinase that acts as a primary transducer of ECM-derived cues [39]. In NPC, ITGB1-mediated FAK activation drives aggressive phenotypes. Our study revealed that rRGD3^mu^ dampens FAK phosphorylation, disrupting the relay of mechanotransductive signals to the downstream AKT pathway. AKT hyperactivation is a major driver of chemoresistance and recurrence in cancer [40,41]. The suppression of p-AKT by rRGD3^mu^ provides a unifying mechanistic explanation for its dual effects: promoting apoptosis by alleviating AKT-mediated inhibition of pro-apoptotic factors while arresting motility by interfering with AKT-dependent cytoskeletal remodeling.

The identification of ITGB1 as a major rRGD3^mu^-associated molecular component provides a plausible explanation for its biological activity. Notably, rRGD3^mu^ induced a dose-dependent reduction in ITGB1 protein abundance. Beyond conventional competitive antagonism, this suggests that rRGD3^mu^ may perturb ITGB1 stability, potentially by triggering ligand-induced endocytosis and subsequent lysosomal degradation [42,43]. While the precise intracellular trafficking pathways and the specific involvement of endocytic mediators warrant further characterization via confocal imaging and biochemical inhibitors [44,45,46], our current data clearly demonstrate that rRGD3^mu^ consistently reduces the availability of ITGB1, thus leading to profound attenuation of the downstream FAK/AKT cascade. Crucially, the functional contribution of ITGB1 was supported by our loss-of-function assays, in which the effects of ITGB1 silencing mimicked those of rRGD3^mu^ and reduced the additional response to rRGD3^mu^ treatment. These findings support an ITGB1-associated mechanism, although additional rescue and direct binding experiments are needed to establish whether ITGB1 is the exclusive or primary functional target. In light of recent genomic characterizations indicating that the CNE2 cell line shares a genetic lineage with HeLa cells, our findings regarding the ITGB1/FAK/AKT axis may reflect a conserved pharmacological vulnerability across integrin-dependent epithelial malignancies rather than a feature strictly restricted to primary nasopharyngeal squamous cells. Nevertheless, the prominent overexpression of ITGB1 in this model aligns with clinical bioinformatic datasets, and the robust, consistent antitumor efficacy observed in our in vivo xenograft model supports the in vivo activity of rRGD3^mu^ in this experimental model and justifies further validation in authenticated NPC systems.

In conclusion, rRGD3^mu^ represents a promising therapeutic innovation that addresses the limitations of traditional RGD-based therapies. Our in vivo efficacy studies demonstrated that, compared with 5-Fu, rRGD3^mu^ inhibited xenograft tumor growth and showed stronger growth-suppressive activity under the tested experimental conditions [47]. Notably, while many RGD-targeted agents focus on inhibiting angiogenesis through other integrin subunits, rRGD3^mu^ exhibits a distinctive functional dependence on ITGB1—a more critical driver of metastasis and chemoresistance in NPC. In the present xenograft experiment, rRGD3^mu^ was tolerated under the tested dosing schedule and inhibited tumor growth. However, these findings should be regarded as preliminary evidence of in vivo efficacy and tolerability rather than definitive evidence of an expanded therapeutic window. Further pharmacokinetic, toxicological, and safety studies are needed.

Although the present data support an important functional contribution of ITGB1-containing integrin complexes to rRGD3^mu^ responses, several methodological and biophysical limitations remain to be addressed in future translational studies. First, regarding peptide characterization, our current quality control relies primarily on tricine-SDS−PAGE profiling; however, the primary band aligns with the theoretical mass, and the faint secondary band below the major band may reflect anomalous electrophoretic mobility of small or highly charged peptides under denaturing conditions; however, minor peptide-related species, degradation products, or residual impurities cannot be excluded by SDS−PAGE alone. Future production and characterization should include MS-based identity confirmation, analytical HPLC-based purity assessment, quantitative endotoxin testing, evaluation of the oligomeric state, and formal stability testing under storage and experimental handling conditions. Second, the precise structural basis governing the rRGD3^mu^/ITGB1 extracellular domain interaction needs full resolution. Future investigations utilizing surface plasmon resonance (SPR) and molecular docking simulations will be pivotal for resolving these binding kinetics [48]. Nevertheless, the results of the present study provide mechanistic evidence supporting the further evaluation of rRGD3^mu^ as a multivalent RGD-based peptide with activity involving ITGB1-containing integrin complexes and the FAK/AKT axis. Additional validation in authenticated NPC models, EBV-positive systems, patient-derived models, and expanded in vivo safety studies will be necessary to determine its broader translational relevance.

## 4. Materials and Methods

### 4.1. Expression and Purification of Recombinant rRGD3^mu^ Peptide

The recombinant rRGD3^mu^ peptide was produced using a bacterial expression system. Briefly, engineered *Escherichia coli* BL21 (DE3) strains harboring the recombinant pET23b-rRGD3^mu^ plasmid (synthesized and constructed commercially) were cultured in LB medium. Peptide expression was initiated by induction with isopropyl-β-D-thiogalactopyranoside (IPTG). The recombinant rRGD3^mu^ peptide was subsequently isolated and refined through Ni-NTA affinity chromatography. Protein quantification was performed using the Bradford assay with Coomassie Brilliant Blue G-250, while the apparent electrophoretic profile and approximate molecular mass were assessed by tricine-SDS−PAGE.

### 4.2. Cell Culture

The CNE2 cell line used in this study was maintained under standard culture conditions. Although genomic characterization studies have reported that CNE2 is a HeLa-derived hybrid lineage [49,50], CNE2 has historically been used as a malignant epithelial cell model in studies related to nasopharyngeal carcinoma and integrin-associated signaling. Therefore, in the present study, CNE2 was used as a CNE2-labeled, ITGB1-expressing malignant epithelial model, with its historical HeLa-derived hybrid origin explicitly acknowledged, rather than as a definitive representative model of nasopharyngeal carcinoma. STR authentication was performed to confirm the identity and support the reproducibility of the cell batch used in this study, but this authentication does not negate the previously reported historical origin of CNE2. CNE2 and HNE1 cells were cultured in RPMI-1640 medium (Gibco, Grand Island, NY, USA) supplemented with 10% fetal bovine serum (FBS) and 1% penicillin−streptomycin, while NP-69 cells were maintained in their designated specialized medium (Anwei Sci, Shanghai, China; Cat# CCLCM-AW-010). All cultures were incubated at 37 °C in a humidified atmosphere containing 5% CO_2_. Prior to experimentation, all cell lines were authenticated, verified as mycoplasma-free, and harvested during the logarithmic growth phase.

### 4.3. Cell Viability Assay

Cell viability was evaluated using the CCK-8 assay. CNE2, HNE1, and NP-69 cells were seeded (5 × 10^3^/well) in 96-well plates and incubated overnight. Cells were then treated with rRGD3^mu^ at specified concentrations for 24, 48, or 72 h. Subsequently, 10 µL of CCK-8 reagent was added to each well, followed by a 1 h incubation at 37 °C. The optical density at 450 nm was measured using a microplate reader (Thermo Fisher Scientific, Waltham, MA, USA). The IC_50_ values were determined through nonlinear regression analysis using GraphPad Prism 10.0 (GraphPad Software, San Diego, CA, USA).

### 4.4. Colony Formation Assay

CNE2 cells (1 × 10^3^/well) were seeded in 6-well plates and incubated overnight. After 24 h of rRGD3^mu^ exposure, the medium was replaced with complete growth medium, and the cells were cultured for an additional 7–14 days. Macroscopic colonies (≥50 cells/colony) were fixed (4% paraformaldehyde, 15 min) and stained (0.1% crystal violet, 20 min). Images were captured and quantified using ImageJ software (version 1.53; National Institutes of Health, Bethesda, MD, USA).

### 4.5. Giemsa Staining Assay

CNE2 cells (1 × 10^5^/well) were seeded in 24-well plates and exposed to rRGD3^mu^ for 24 h. The cells were then fixed in methanol (15 min) and stained with Giemsa solution (20 min) following the manufacturer’s protocols. After the cells were rinsed and air-dried, their morphology and density were assessed using an inverted light microscope (Olympus, Hachioji, Japan). Representative photomicrographs were captured to illustrate the observed phenotypic variations.

### 4.6. Migration Assay

CNE2 cells (2.5 × 10^5^ cells/mL) in serum-free RPMI-1640 supplemented with rRGD3^mu^ were seeded into the upper Transwell chamber. The lower chamber contained RPMI-1640 with 10% FBS. After 24 h, nonmigrated cells were removed. The cells that migrated to the lower membrane were fixed (4% paraformaldehyde, 15 min), stained (0.1% crystal violet, 20 min), and imaged. The migratory capacity was quantified by counting the number of cells per membrane.

### 4.7. Invasion Assay

Cell invasion was assessed using Transwell inserts precoated with 80 µL of Matrigel (1:7 dilution; Corning, Corning, NY, USA). After solidification at 37 °C, CNE2 cells (5 × 10^5^ cells/mL) resuspended in serum-free medium containing rRGD3^mu^ were added to the upper chamber (200 µL/well). The lower chamber contained 700 µL of RPMI-1640 supplemented with 10% FBS. After 24 h, noninvading cells and Matrigel were gently removed. Invaded cells were fixed (4% paraformaldehyde, 15 min), stained (0.1% crystal violet, 20 min), and imaged. The invasion potential was quantified by counting the mean cell number per field.

### 4.8. Adhesion Assay

First, 96-well plates were precoated with 0.1 µg/µL of vitronectin (VN), fibronectin (FN), or laminin (LN) (2 h, 37 °C). CNE2 cells (2 × 10^4^/well) in serum-free medium containing rRGD3^mu^ were seeded. After 4 h, the nonadherent cells were removed by rinsing with PBS. Adhesion efficiency was assessed using a CCK-8 assay; the absorbance (450 nm) was measured and normalized to that of the control group. All assays were performed in triplicate.

### 4.9. TUNEL Assay

CNE2 cells were seeded in 24-well plates and exposed to rRGD3^mu^ at 50% confluence. After 24 h, the cells were fixed (4% paraformaldehyde, 15 min) and permeabilized (0.1% Triton X-100, 10 min). Apoptosis was visualized using a TUNEL assay (37 °C, 1 h, dark) per the manufacturer’s instructions. The nuclei were counterstained with DAPI (5 min). The fluorescent signals were imaged via an inverted fluorescence microscope (Olympus, Japan).

### 4.10. Animal Experiments

Animal protocols were approved by the Dalian Medical University Ethics Committee (AEE24326). Male BALB/c nude mice (4–6 weeks old) were subcutaneously injected with CNE2 cells (5 × 10^6^ in 100 µL of PBS) in the right axilla. After palpable tumor formation (10–14 days), the mice were randomized into five cohorts (n = 6): saline control, rRGD3^mu^ (25, 50, and 100 µg/kg), and 5-Fu (10 mg/kg). No animals were excluded from the analysis. Treatments were administered i.p. for 14 consecutive days (rRGD3^mu^: BID; 5-Fu: QD). The investigators were blinded to group allocation during tumor measurement and data analysis. Body weight and tumor dimensions (length a and width b) were recorded every two days. Tumor volume (V) was calculated as V = 1/2 × a × b^2^. Following the final treatment, the mice were euthanized, and the tumors were excised, weighed, and photographed.

Animal housing and experimental procedures were performed in accordance with the ARRIVE guidelines and the 3Rs principles to minimize animal suffering. The mice were maintained under SPF conditions (12 h light/dark cycle; ad libitum access to food and water).

### 4.11. H&E Staining

The tumor tissues were paraffin-embedded and sliced (4 µm). After deparaffinization and rehydration in graded ethanol, the sections were H&E stained. The slides were dehydrated, cleared, and mounted. Representative images were acquired via light microscopy (Olympus, Japan) to evaluate tissue architecture.

### 4.12. Western Blot

Total protein was extracted using IP lysis buffer containing protease and phosphatase inhibitors and then quantified via a BCA assay (Beyotime, Shanghai, China). Protein samples (10%/12% SDS−PAGE) were resolved and electrotransferred onto PVDF membranes. Following blocking with 5% nonfat milk (2 h), the membranes were incubated overnight (4 °C) with primary antibodies (all 1:1000): Bax, caspase-9, cleaved caspase-9, caspase-3, N-cadherin, E-cadherin, vimentin, MMP2, FAK, p-FAK, AKT, p-AKT (Proteintech, Wuhan, China), Bcl-2, cleaved caspase-3 (Selleck, Houston, TX, USA), and GAPDH (Boster, Wuhan, China). After HRP secondary antibody incubation (1:5000, 1 h), signals were detected using ECL (Beyotime) and imaged (Amersham Imager 600 GE Healthcare Bio-Sciences AB, Uppsala, Sweden). GAPDH served as the loading control.

### 4.13. Cellular Thermal Shift Assay (CETSA)

CNE2 cells were treated with rRGD3^mu^ or vehicle for 2 h, harvested, and resuspended in protease inhibitor-supplemented PBS. Aliquots were distributed into PCR tubes and subjected to a 57–84 °C temperature gradient for 3 min using a thermal cycler, followed by 3 min at room temperature. Cell lysis was achieved via three freeze−thaw cycles in liquid nitrogen. The soluble fraction was harvested by centrifugation (12,000× *g*, 20 min, 4 °C), and the supernatant was analyzed via Western blotting to evaluate the thermal stability of ITGB1.

### 4.14. siRNA Transfection

Cells were seeded in 6-well plates and grown to 60% confluence prior to transfection. To establish appropriate baselines, cells were assigned to a nontransfected (Blank) group, a negative control (si-Control) group, or groups transfected with one of two independent ITGB1-specific siRNAs (si-ITGB1-1: 5′-GGUAGAAAGUCGGGACAAATT-3′; si-ITGB1-2: 5′-GCGAGTGTGATAATTTCAA-3′). Transfections were performed using Lipofectamine 3000 (Invitrogen, Carlsbad, CA, USA) at a final siRNA concentration of 30 nM in accordance with the manufacturer’s instructions. Following a 6–8 h incubation, the transfection medium was replenished with fresh complete medium. Knockdown efficiency was validated via Western blotting at 48 h post-transfection.

### 4.15. Statistical Analysis

The data are expressed as the means ± SDs (n ≥ 3 independent experiments). The normality of the data distribution was assessed using the Shapiro–Wilk test, and the homogeneity of variance was confirmed via the Brown–Forsythe test. Statistical differences were analyzed via Student’s *t* test (two groups) or one-way ANOVA followed by Tukey’s post hoc test (multiple groups) using GraphPad Prism 10.0. Significance was defined as *p* < 0.05 (* *p* < 0.05, ** *p* < 0.01).

## 5. Patents

The work reported in this manuscript has been filed as a Chinese invention patent (Application No. 202510504450.2).

## Data Availability

The original contributions presented in this study are included in the article/Supplementary Material. Further inquiries can be directed to the corresponding authors.

## Supplementary Materials

The following supporting information can be downloaded at https://www.mdpi.com/article/10.3390/ijms27115045/s1.
