# Supplementary material for: rRGD3mu, a Triple-RGD Recombinant Peptide, Suppresses Malignant Phenotypes in Nasopharyngeal Carcinoma-Associated Models Through the Modulation of ITGB1-Associated FAK/AKT Signaling
Source: Int J Mol Sci. 2026 Jun 3;27(11):5045. doi: 10.3390/ijms27115045 (PMC13256499; doi:10.3390/ijms27115045)
Supplement: Supplementary file 1 [file ijms-27-05045-s001.zip › ijms-4305304-supplementary.pdf]

Cell Line Authentication Service

STR Profiling Report

Sample Code: CNE-2

Sample Type: Cell Line

Testing Method: STR Genotyping

Report Time: May 15, 2024

Add: Room 502, NO.1015 Longteng Rd , Songjiang District, Shanghai/ Tel:+86-18521538068 / E-mail: zhusn@biowing.com.cn

COMPANY STATEMENT

1. THIS REPORT IS ONLY RESPONSIBLE FOR THE SAMPLES ANALYZED.
2. THE TESTING RESULTS AND THE ORGANIZATION NAME WILL NOT BE USED FOR ADVERTISEMENT, COMMERCIAL EXHIBITIONS, COMMERCIAL PERFORMANCE AND OTHER COMMERCIAL ACTIVITIES.
3. OBJECTIONS SHOULD BE RAISED WITHIN FIFTEEN DAYS AFTER THE RECEIPT OF THIS REPORT.
4. THE PAPER REPORT WITH CONTENT ALTERING, ADDING OR WITHOUT THE STAMPED SEAL OF THE COMPANY ARE INVALID.

Testing Company: Shanghai Biowing Applied Biotechnology Co. Ltd
Address: Room 502, NO.1015 Longteng Rd , Songjiang District, Shanghai
Tel: +86-18521538068
Contact: Shuangning Zhu
E-mail: zhusn@biowing.com.cn

Cell Line Authentication – STR Profiling Report

Sample code

Table S1. Sample Code

Customer's code	Company Code
CNE-2	20240510-01

Sample Number :1

Sample Type: Cell line

Testing Type: STR

Testing Method:

In accordance with the national standard **GA/T 1163-2014** *Analysis and Application of Human DNA Fluorescent STR Typing Results*, genomic DNA was extracted from the sample using a commercial DNA extraction kit (CORNING, Cat. No. AP-EMN-BL-GDNA-250G). Multiplex PCR amplification was performed for a total of twenty-one STR loci including the Amelogenin locus. The amplified products were separated and detected on an ABI 3730XL Genetic Analyzer (Thermo Fisher Scientific). The raw signal data generated by the analyzer was further analyzed and interpreted using GeneMapper software to obtain the final genotyping results.

Data Interpretation:

The STR profile database used in this test complies with the international cell bank official standard **ANSI/ATCC ASN-0002-2021** *Authentication of Human Cell Lines: Standardization Of Short Tandem Repeat (STR) Profiling* (ANSI eStandards Store, 2021). This standard was jointly developed by an international expert working group (ASN-002) under the auspices of an ANSI-accredited Standard Development Organization (SDO) led by the American Type Culture Collection (ATCC).

Add: Room 502, NO.1015 Longteng Rd , Songjiang District, Shanghai/ Tel:+86-18521538068
/ E-mail: zhusn@biowing.com.cn

Test Results

1. STR profile

Table S2. STR and Amelogenin Genotyping Results of Cell line.

Loci	Sample information			Cell Bank information		
	Sample name: CNE-2			Cell line name: CNE-2		
	**Allele1**	**Allele2**	**Allele3**	**Allele1**	**Allele2**	**Allele3**
D5S818	11	12		11	12	
D13S317	10	12	13.3	10	12	13.3
D7S820	10	12		10	12	
D16S539	9	10		9	10	
VWA	14	16	17	14	16	
TH01	6	7	9	6	7	9
AMEL	X	X		X	X	
TPOX	8	9		8	9	12
CSF1PO	10	11		10	11	
D12S391	20	21				
FGA	18	21				
D2S1338	17	23				
D21S11	27	30				
D18S51	13	15				
D8S1179	12	13	17			
D3S1358	15	18				
D6S1043	11	14	18			
PENTAE	17	20				
D19S433	13	13				
PENTAD	9	12				
D1S1656	12	15				

Add: Room 502, NO.1015 Longteng Rd , Songjiang District, Shanghai/ Tel:+86-18521538068 / E-mail: zhusn@biowing.com.cn

2. database annotation

Figure S1. STR matching analysis

EV	Cell No.	Cell name	Locus names								
			D5S818	D13S317	D7S820	D16S539	VWA	TH01	AM	TPOX	CSF1PO
	Query (Your Cell)		11,12,	10,12,13.3	10,12,	9,10,	14,16,17	6,7,9	X,X,	8,9,	10,11,
0.95(34/36)	CVCL_6889	CNE-2	['11', '12']	['10', '12', '13.3']	['10', '12']	['9', '10']	['14', '16']	['6', '7', '9']	['X', 'X']	['8', '9', '12']	['10', '11']

Note: The STR online match analysis of the test cell against DSMZ/ATCC/EXPASY database, showing cell number (Cell No.) and cell name.

3. Authentication

☐ The submitted sample profile is human, but not a match for any profile in the DSMZ STR database.

☑ The submitted profile is basic match for the following human cell line(s) in the DSMZ STR database (8 core loci plus Amelogenin): **CNE-2**.

☐ The submitted profile is similar to the following DSMZ human cell line: /.

- **Note:** Cell lines are considered to related, derived from a common ancestry, when EV≥80% of the alleles in its STR profile match profiles from tissue or other cell line samples from that donor or from database. Cell lines with between an EV match of 60%-80% is unlikely to come from the same donor, requires further verification to confirm its relevance; an EV match of ≤59% comes from a different donor.

4.Genotyping Strategy and Site Distribution:

Table S3. Experimental Strategy and Sites

	Strategy 1	Strategy 2	Strategy 3	Strategy 4
1	D3S1358	D8S1179	D19S433	AMEL
2	VWA	D21S11	TH01	D1S1656
3	D7S820	D16S539	D13S317	D5S818
4	CSF1PO	D2S1338	TPOX	D12S391
5	PENTAE	PENTAD	D18S51	FGA
6			D6S1043	

Shanghai Biowing Applied Biotechnology Co. Ltd
STR Profiling
Cell Line Authentication

Technician: Xiuchuan He

Checked by: Chenqian Zhang

Issued by: Min Wang

Issue date: November 15, 2024

Add: Room 502, NO.1015 Longteng Rd , Songjiang District, Shanghai/ Tel:+86-18521538068 / E-mail: zhusn@biowing.com.cn

Figure S2. STR profiles of sample cell line

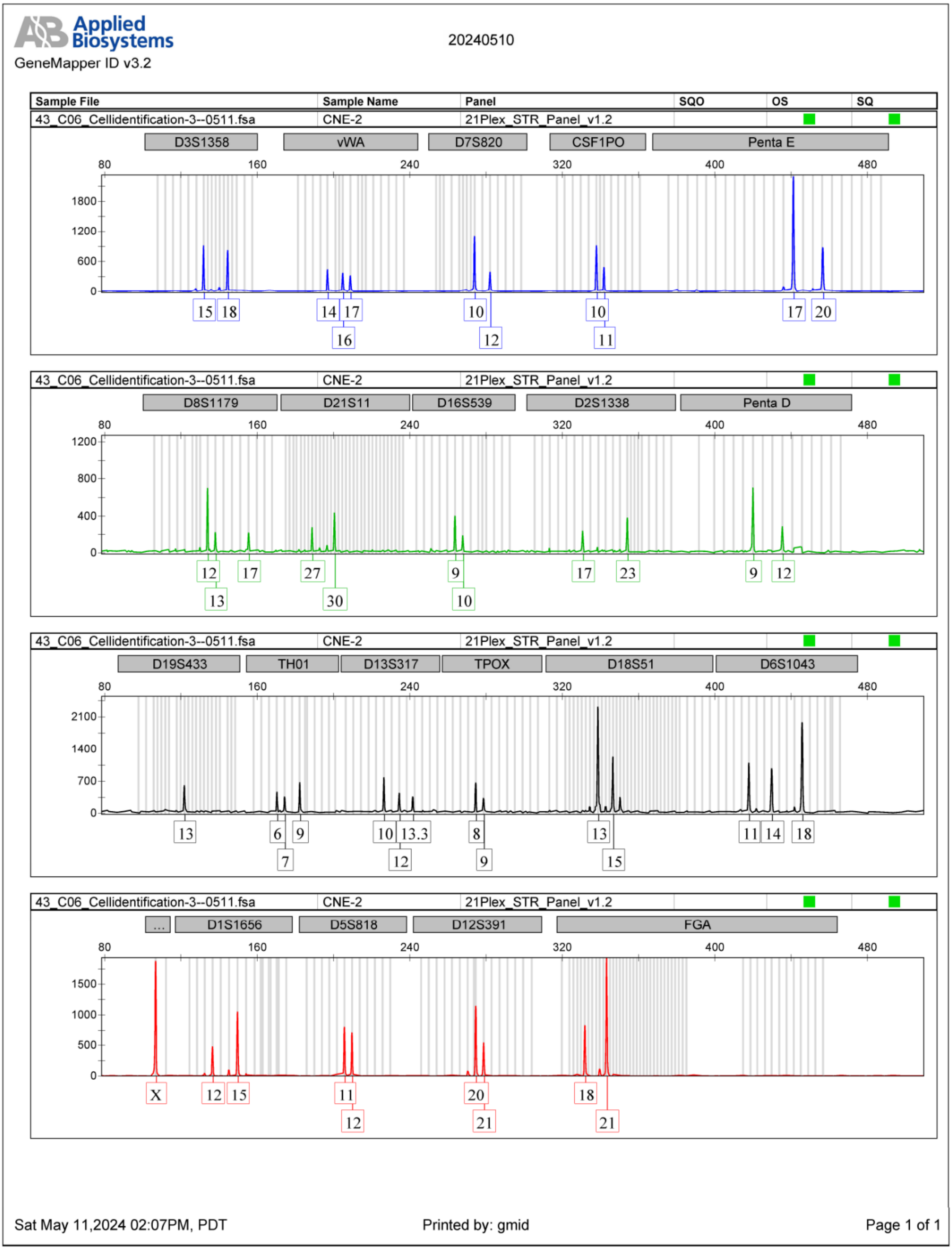

Add: Room 502, NO.1015 Longteng Rd , Songjiang District, Shanghai/ Tel:+86-18521538068 / E-mail: zhusn@biowing.com.cn
